# Identification of Environmental Factors Associated with Inflammatory Bowel Disease in a Southwestern Highland Region of China: A Nested Case-Control Study

**DOI:** 10.1371/journal.pone.0153524

**Published:** 2016-04-12

**Authors:** Junkun Niu, Jiarong Miao, Yuan Tang, Qiong Nan, Yan Liu, Gang Yang, Xiangqian Dong, Qi Huang, Shuxian Xia, Kunhua Wang, Yinglei Miao

**Affiliations:** 1 Department of Gastroenterology, The First Affiliated Hospital of Kunming Medical University, Yunnan Institute of Digestive Disease, Kunming, Yunnan Province, P. R. China; 2 Department of Gastroenterology, The First People's Hospital of Qujing, Qujing, Yunnan Province, P. R. China; 3 Department of General Surgery, The First Affiliated Hospital of Kunming Medical University, Yunnan Institute of Digestive Disease, Kunming, Yunnan Province, P. R. China; University Hospital Llandough, UNITED KINGDOM

## Abstract

**Background:**

The aim of this study was to examine environmental factors associated with inflammatory bowel disease (IBD) in Yunnan Province, a southwestern highland region of China.

**Methods:**

In this nested case-control study, newly diagnosed ulcerative colitis (UC) cases in 2 cities in Yunnan Province and Crohn’s disease (CD) cases in 16 cities in Yunnan Province were recruited between 2008 and 2013. Controls were matched by geography, sex and age at a ratio of 1:4. Data were collected using the designed questionnaire. Conditional logistic regression models were used to estimate adjusted odds ratios (ORs).

**Results:**

A total of 678 UC and 102 CD cases were recruited. For UC, various factors were associated with an increased risk of developing UC: dietary habits, including frequent irregular meal times; consumption of fried foods, salty foods and frozen dinners; childhood factors, including intestinal infectious diseases and frequent use of antibiotics; and other factors, such as mental labor, high work stress, use of non-aspirin non-steroidal anti-inflammatory drugs and allergies (OR > 1, *p* < 0.05). Other factors showed a protective effect: such as consumption of fruits, current smoking, physical activity, and drinking tea (OR < 1, *p* < 0.05). For CD, appendectomy and irregular meal times increased the disease risk (OR >1, *p* < 0.05), whereas physical activity may have reduced this risk (OR < 1, *p* < 0.05).

**Conclusions:**

This study is the first nested case-control study to analyze the association between environmental factors and IBD onset in a southwestern highland region of China. Certain dietary habits, lifestyles, allergies and childhood factors may play important roles in IBD, particularly UC.

## Introduction

Inflammatory bowel disease (IBD) includes ulcerative colitis (UC) and Crohn’s disease (CD), and its etiology and pathogenesis remain unclear. Studies have shown that genetics, the external environment and the gut microbiome play important roles in the development of IBD. Over recent decades, the incidence and prevalence of IBD have increased dramatically, especially in previously low-incidence Asian regions [1]. A two- to three-fold increase in the incidence of IBD has been noted in several countries in Asia [2]. The rather quick change in IBD incidence strongly suggests that environmental factors have a clear etiological role in IBD [3]. In particular, evidence suggests that consumption of a Western diet enriched with saturated fat, refined carbohydrates, and food additives is associated with increased IBD risk [4].

The literature is inundated with environmental risk-factor studies from the Western world, but analogous studies in Asia, especially in China, are scarce. Although the ACCES study included three cities in China [5], the economic level of these cities is more developed and does not overlap with the region investigated in our study.

Here, we report the results of the first nested case-control study in Yunnan Province, which is located in the less-developed, southwestern highland region of China where IBD is emerging. Thus, this represents an ideal time and place to study the environmental determinates of IBD.

## Methods

### Cohort population

In phase 1 of this study, we established the network system of IBD: There are 16 cities in Yunnan Province. We established 1 to 3 surveillance points in each city and the network management system for newly diagnosed IBD patients prior to January 1, 2008. The 34 surveillance points utilized in this study were all located in comprehensive hospitals in Yunnan Province. One or two physicians from each collaborating hospital were designated and specially trained as coordinators. These designated investigators recruited newly diagnosed IBD cases and referred suspected cases to the gastroenterologists in our research group for definite diagnosis. Every 3 to 6 months, these investigators visited local inpatient wards, endoscopy centers, outpatient clinics, and community clinics to identify IBD cases, and this process was supported by the local hospitals and communities. To ensure completeness of case capture, a multifaceted approach that included advertisements in local media and regular health education lectures at 34 surveillance points was used to identify cases of IBD.

In Yunnan Province, a total of 1,281 newly diagnosed UC patients and 59 CD patients were enrolled into the network management system between 1998 and 2007. Combined with Yunnan Province demographic data, the crude incidence rate of UC and CD was 0.428 and 0.029 per 100,000 individuals, respectively, in 2007, as reported in another paper (data not yet published).

Phase 2–cohort population ascertainment: Based on data from the network management system, we initiated another component regarding environmental factors of IBD from January 2008 to December 2013. IBD screening was performed in Yunnan Province before January 1, 2008. The established UC and CD cases were excluded from the cohort population. Before the study was conducted, we completely estimated the sample size. The incidence rates of IBD in Kunming and Qujing cities rank among the highest in Yunnan Province, and the UC samples in these groups may meet the requirement for statistical analysis. Thus, we selected permanent residents under surveillance in these two cities as the cohort population of UC. However, the sample size of CD cases from these two cities was less than the number of UC cases. To increase the sample size of CD cases, the cohort population included permanent residents of Kunming, Qujing and 14 other cities in Yunnan Province. The permanent residents included both native inhabitants and temporary residents who lived in Yunnan Province for ≥ 6 months between January 1, 2008 and December 31, 2013.

### Cases and controls

Newly diagnosed cases that met the diagnostic criteria for IBD were registered in the network management system by the designated investigators at 34 surveillance points in Yunnan Province. The eligible patients were divided into two groups (UC group and CD group) between January 1, 2008 and December 31, 2013. IBD patients were confirmed according to the Lennard-Jones criteria [6]. The disease phenotype was measured according to the Montreal classification [7].

The designated gastroenterologists of our research group and two pathologists were invited to make a definite diagnosis. In the corresponding cohort population, once we diagnosed an IBD case, healthy subjects in the matched communities were matched by age (±3 years) and sex at a ratio of 1:4 (case-control) and selected as controls (UC control group and CD control group). Patients that did not meet the diagnostic criteria for IBD and IBD-undetermined cases were excluded. Patients that refused to participate in our study and patients less than 14 years old or had severe multi-system diseases were also excluded.

The study was approved by the Ethics Committee of Kunming Medical University. Written informed consent was obtained from all participants or their guardians (younger than age 18).

### Environment exposures

The questionnaire (S1 File) was designed by our research group organizer, a gastroenterologist with an interest in IBD, by combining relevant epidemiologic studies [8–9] and under the guidance of epidemiology specialists. In total, approximately 40 items were included in the questionnaire; some questions were previously used in other studies of IBD [8–9]. Participants were encouraged to corroborate answers. Wherever possible, data were recorded categorically using ‘yes’, ‘no’, and ‘unsure’ answers to reduce information bias associated with the need to answer difficult questions. The questionnaire included the following five main areas: (1) General states, including sex (male, female), nationality (Han, minority), education level (primary school, secondary school, university), and labor type (manual labor, mixed, mental labor). Individuals who perform manual labor include unskilled manual workers, such as farm and forestry workers. Individuals who perform mental labor include individuals performing desk jobs, such as clerical workers, managers, and scientific research-related workers, and mixed labor includes some skilled and specialized workers involved in more manual labor, such as drivers and seamen. (2) Childhood (younger than 14 years) factors, including delivery mode (natural birth, cesarean), childhood intestinal infections (never, 1–2 times/year, ≥ 3 times/year), and breast-feeding (never, < 3 months, ≥ 3 months). (3) Medication use, including non-aspirin non-steroidal anti-inflammatory drugs (NA-NSAIDs, < 1 month, ≥ 1 month) before diagnosis (including indomethacin, diclofenac and others) and oral contraceptive (OCP) use (never, past, current < 5 years, current ≥ 5 years). (4) Dietary habits and life style factors, such as smoking (current smoking, ex-smoking, never smoking), drinking (never, yes), consumption of tea (no, yes), physical activity and mean sleep duration. Dietary habits were defined as typical intake over 1 month. These habits were categorized into three groups [frequent (≥ 3 times/week), moderate (1–2 times/week) and never] regarding the consumption of fruits, vegetables, eggs, meat, sugars and sweets, fish, fried foods, salty foods, frozen dinners, and spicy foods; irregular meal times were also noted. Current smoking was defined as smoking at least one cigarette/day (daily dose of cigarette exposures: < 10 cigarettes, 10–20 cigarettes, > 20 cigarettes). Ex-smoking was used to indicate those reporting having smoked at least one cigarette/day, and never smoking referred to those who never smoked one cigarette/day. Drinking was defined as more than one alcoholic drink per month. Physical activity was defined as any rhythmic and continuous activity performed for greater than 20 minutes at a time. (5) Other factors included appendectomy, pet ownership, allergies, and parasitic infections.

A simulated investigation was tested in 10 IBD patients and 10 controls. Data were processed, validated, and verified between January 1, 2014 and December 31, 2014. When we diagnosed an IBD case, one or two designated investigators from each collaborating hospital sent the questionnaire to the patient. The participant completed the questionnaire with the investigator’s guidance to insure the authenticity and integrity of the data. The items were logged in the network management system. Every three months, the designated investigators visited the local communities. The healthy subjects were interviewed face-to-face. The designated investigators completed the questionnaires as quickly and reliably as possible.

### Statistical analysis

Each investigator coded most of the answers for computer analysis using standard rules. SPSS 19.0 statistical software package (SPSS Inc., Chicago, IL, USA) was used for analysis. Separate analyses were performed for UC cases and their matched controls and for CD cases and their matched controls. Descriptive variables are presented as medians (interquartile range, IQR), and categorical variables are presented as frequencies with percentages. Each environmental factor was first tested by univariate analysis with odds ratios (OR) and 95% confidence intervals (95% CI). In multivariate analysis, based on conditional logistic regression, variables with a *p*-value < 0.05 in the univariate analyses were proposed for entry into the model. Rates of ‘unsure’ responses were compared between cases and controls to assess for differential reporting. A *p*-value of < 0.05 was considered significant.

## Results

Between January 1, 2008 and December 31, 2013, after the exclusion of 57 UC and 35 CD patients who were not eligible for the case group, 678 UC cases and 102 CD cases were recruited. IBD-undetermined cases represented less than 3% of IBD patients and were excluded from our research. The response rate was 92.2% for UC and 74.5% for CD patients. Excluding those not cooperative in answering some questions, 2,712 and 410 matched controls for UC and CD, respectively, were included in this study. The response rates for the UC and CD control groups were 81.1% and 77.3%, respectively.

The UC patient cohort included 372 (54.9%) males and 306 (45.1%) females. The median age was 46.45 ± 15.74 years and 46.37 ± 15.60 years for UC cases and controls, respectively. The CD patient cohort included 66 (64.7%) males and 36 (35.3%) females. The median age of CD cases and controls was 39.03 ± 16.26 years and 38.53 ± 15.92 years, respectively. Subjects in the case and control groups were matched by sex and age with no significant differences (*t* = 0.119, *p* = 0.906; *t* = 0.281, *p* = 0.779, respectively, for UC and CD). The phenotype of IBD patients is presented in Table 1. In CD, the L4 phenotype was noted in 3 patients, 1 with colonic disease and 2 with ileocolonic disease. No isolated upper gastrointestinal tract disease was noted.

**Table 1:** Disease phenotype of IBD patients.

Disease phenotype	UC, n = 678 (%)	CD, n = 102 (%)
**Sex**:		
**Male**	372 (54.9)	66 (64.7)
Female	306 (45.1)	36 (35.3)
Male/Female	1.21/1	1.83/1
**Median age at diagnosis**:		
(y, mean ± SD)	46.5 ± 15.7	39.0 ± 16.3
**Location of disease (UC)**:		
E1—Proctitis	112 (16.5)	**–**
E2—Left-sided colitis	335 (49.4)	**–**
E3—Extensive/pancolitis	231 (34.1)	**–**
**Location of disease (CD)**:		
L1—Terminal ileum	**–**	36 (35.3)
L2—Colon	**–**	13 (12.7)
L3—Ileocolon	**–**	53 (52.0)
L4—Upper gastrointestinal	**–**	3 (2.9)
**Disease behavior (CD)**:		
B1—Inflammatory	**–**	54 (52.9)
B2—Stricturing	**–**	31 (30.4)
B3—Penetrating	**–**	17 (16.7)
P—Perianal disease	**–**	3 (2.9)

### Univariate analysis of UC cases and controls

In the univariate analysis of UC patients and matched controls, statistically significant (*p* < 0.05) results were obtained for 16 items (S1 Table). Factors such as allergies, childhood antibiotic use (≥ 3 times/year), childhood intestinal infections (1–2 times/year), previous NA-NSAIDs intake (within 1 month), mental labor, high work stress, mean sleep duration < 6 hours, frequent irregular meal times, and consumption of fried foods, salty foods, and frozen dinners were associated with a higher proportion of UC patients compared with controls (OR > 1). However, the consumption of tea and fruits, current smoking, and physical activity were more frequently noted in controls compared with cases (OR < 1). Different types of drinking water may also be associated with UC. No significant differences between cases and controls were observed regarding breast-feeding, OCP use and drinking.

### Univariate analysis of CD cases and controls

In the univariate analysis of CD and matched controls, statistically significant (*p* < 0.05) results were obtained for the following five factors: allergies, previous appendectomy, physical activity, frequent irregular meal times and consumption of vegetables. Among the CD cases and controls, none showed a family history of disease or OCP use (S2 Table).

### Multivariate analysis of UC cases and matched controls

Statistically significantly different factors between UC cases and controls in the univariate analysis were subjected to multivariate conditional logistic regression analysis, which demonstrated that the following factors were associated with an increased risk of developing UC: some dietary habits, including frequent irregular meal times (OR: 2.287; 95% CI: 1.494–3.825), eating fried foods (OR: 1.920; 95% CI: 1.253–3.254), salty foods (OR: 1.465; 95% CI: 1.046–2.726) and frozen dinners (OR: 1.868; 95% CI: 1.392–2.854); childhood factors, including intestinal infectious diseases (1–2 times/year, OR: 1.836; 95% CI: 1.182–2.641); and other factors, such as mental labor (OR: 2.013; 95% CI: 1.414–3.264), high work stress (OR: 1.732; 95% CI: 1.142–2.628), previous NA-NSAIDs intake (within 1 month, OR: 2.893; 95% CI: 1.619–5.312) and allergies (OR: 5.361; 95% CI: 2.469–11.639). Other factors were suggested as protective factors against UC: consumption of fruits (1–2/week, OR: 0.498, 95% CI: 0.282–0.801; ≥ 3 times/week, OR: 0.423, 95% CI: 0.254–0.773), current smoking (OR: 0.409; 95% CI: 0.313–0.639), physical activity (1–2/week, OR: 0.655, 95% CI: 0.391–0.788; ≥ 3 times/week, OR: 0.461, 95% CI: 0.319–0.672), and consumption of tea (OR: 0.338, 95% CI: 0.275–0.488). Different types of drinking water exhibited no statistically significant difference in multivariate analysis. However, because only 5 UC cases and no CD cases had a positive family history of disease, the item “family history” was excluded from the data analysis (Table 2).

**Table 2:** Multivariate logistic regression analysis of UC patients and controls.

UC variables	*p*	OR	95% CIs
**Allergies**:	**<0.001**	**5.361**	**2.469–11.639**
**Childhood antibiotic use**:			
1–2 times /year	0.202	0.628	0.308–1.282
≥3 times/year	**0.024**	**2.364**	**1.432–3.572**
**Childhood intestinal infections**:			
1–2 times /year	**0.011**	**1.836**	**1.182–2.641**
≥ 3 times/year	0.316	0.819	0.527–1.462
**NA-NSAIDs intake**:			
< 1 month	**0.001**	**2.893**	**1.619–5.312**
≥ 1 month	0.082	1.327	0.827–3.125
**Labor type**:			
Mixed labor	0.260	1.293	0.716–1.850
Mental labor	**<0.001**	**2.013**	**1.414–3.264**
**Work stress**:			
General	0.570	1.235	0.749–1.743
High	**0.022**	**1.732**	**1.142–2.628**
**Irregular meal times**:			
1–2 times /week	0.338	0.805	0.516–1.255
≥ 3 times /week	**<0.001**	**2.287**	**1.494–3.825**
**Consumption of fried foods**:			
1–2 times /week	0.582	1.272	0.849–1.585
≥ 3 times /week	**0.004**	**1.920**	**1.253–3.254**
**Consumption of salty foods**:			
1–2 times /week	0.446	0.868	0.602–1.250
≥ 3 times /week	**0.022**	**1.465**	**1.046–2.726**
**Frozen dinners**:			
1–2 times /week	0.082	1.424	0.892–2.158
≥ 3 times /week	**0.002**	**1.868**	**1.392–2.854**
**Drinking water**:			
Well water-based (%)			
Tap water-based (%)	0.999	0.003	0.001–0.101
Boiled water-based (%)	0.361	0.433	0.072–2.606
Mineral water-based (%)	0.100	0.220	0.036–1.338
**Consumption of fruits**:			
1–2 times /week	**0.007**	**0.498**	**0.282–0.801**
≥ 3 times /week	**0.001**	**0.423**	**0.254–0.773**
**Tea consumption**:	**<0.001**	**0.338**	**0.275–0.488**
**Current smoking**:	**0.030**	**0.409**	**0.313–0.639**
Ex-smoking	0.063	1.341	0.851–3.281
**Physical activity**:			
1–2 times /week	**<0.001**	**0.655**	**0.391–0.788**
≥ 3 times /week	**<0.001**	**0.461**	**0.319–0.672**
**Sleep duration ≥ 6 hours**:	0.072	0.810	0.612–1.392

Statistically significant (*p* < 0.05) results are shown in **bold**. CI, confidence interval; OR, odds ratio.

### Multivariate analysis of CD cases and controls

Similarly, multivariate conditional logistic regression analysis revealed that prior appendectomy (OR: 2.848; 95% CI: 1.444–4.217) and frequent irregular meal times (≥ 3 times/week, OR: 1.876; 95% CI: 1.807–3.236) may increase the risk of developing CD. In contrast, physical activity (1–2 times/week, OR: 0.332, 95% CI: 0.131–0.937; ≥ 3 times/week, OR: 0.505, 95% CI: 0.217–1.090) may represent a protective factor against disease development. Allergies and the consumption of vegetables, which were statistically significant (*p* < 0.05) in univariate analyses, did not remain statistically significant in the multivariate analysis (Table 3).

**Table 3:** Multivariate logistic regression analysis of CD patients and controls.

CD variables	*p*	OR	95% CIs
**Allergies**:	0.137	1.770	0.833–3.758
**Appendectomy**:	**0.024**	**2.848**	**1.444–4.217**
**Irregular meal times**:			
1–2 times /week	0.216	1.480	0.795–2.752
≥ 3 times /week	**0.024**	**1.876**	**1.807–3.236**
**Consumption of vegetables**:			
≥3 times /week	0.081	0.597	0.335–1.607
**Physical activity**:			
1–2 times /week	**0.000**	**0.332**	**0.131–0.937**
≥ 3 times /week	**0.014**	**0.505**	**0.217–1.090**

Statistically significant (*p* < 0.05) results are indicate in **bold**. CI, confidence interval; OR, odds ratio.

## Discussion

This nested case-control study of environmental factors associated with IBD in a less-developed, southwestern highland region of China confirmed the relation between the development of IBD and certain established risk factors and revealed some novel associations.

Cigarette smoking is the most widely and consistently described environmental factor associated with IBD. For UC, most studies, including trials in New Zealand [10–11], Spain [12] and the United States [13], indicate that smoking is a protective factor in the development of UC. However, smoking increases the risk of developing CD, as noted by data from the United States [13] and Canada [8]. Smoking may have a suppressive effect on T cells, resulting in an altered intestinal microbiome [14]. For Asians, smoking was not a significant risk factor for CD, and previous history of smoking increased the risk of developing UC [5]. Our study confirmed the negative association of current smoking at diagnosis with UC (OR: 0.409) but not CD. The reason for these different effects may be attributed to the limited amount of data on CD subjects in our study or may be because smoking does not play the same role in different ethnic groups.

The relationship between appendectomy and IBD remains unclear. Data from most Western and Asian countries indicate that previous appendectomy is protective for the development of UC but not the risk for CD, and these data are similar to those reported in Spain [12], Australia [15] and Japan [16]. However, Siew et al. reported that appendectomy did not influence the risk of IBD in Asians [5]. Recently, a cohort study demonstrated that during childhood or adolescence, appendicitis and mesenteric lymphadenitis, but not appendectomy itself, are associated with a significantly reduced risk of developing UC [17]. For CD, a meta-analysis conducted in Canada revealed that the risk of CD increases significantly within 4 years after appendectomy (RR 1.99, 95% CI 1.66–2.38); however, after 5 years or more, the risk is reduced to baseline levels (RR 1.08, 95% CI 0.99–1.18), thus indicating diagnostic problems in patients with incipient CD [18]. Our study demonstrated that although previous appendectomy was not correlated with the development of UC, it increased the risk of developing CD by 2.85-fold compared with patients without appendectomy (OR: 2.848). However, we cannot exclude the possibility that some of the CD cases may have been misdiagnosed, as this condition presents with symptoms similar to those of appendicitis. The role of appendectomy in the pathogenesis of IBD is complex. Some researchers suggest that the appendix plays a role in regulating the intestinal microbiota, and alterations in mucosal immune responses leading to appendicitis or resulting from appendectomy may negatively affect the pathogenesis of UC [19].

Infancy and childhood factors include intestinal infections, antibiotic use, and breast-feeding, although evidence regarding the association between enteric infections and the risk of developing IBD is insufficient [11]. A study conducted in Spain revealed that respiratory and enteric infections are protective factors against IBD in children [12]. However, another study revealed that acute gastrointestinal infections are a risk factor, particularly infections in patients 1 year of age [20]. Unlike previous data, Siew et al. [5] reported no associations between any of the childhood infections and the risk of developing CD or UC. Breast-feeding and the duration-response protective factor for the subsequent development of IBD were demonstrated in New Zealand [10], and another case-control study reported that the protective effect of breast-feeding was only significant when the duration of breast-feeding was greater than 12 months in UC and CD [5]. Our study concluded that childhood intestinal infections may positively affect the development of UC but not CD. Frequent antibiotic use during childhood was more common in UC cases compared with controls, whereas it had no effect on CD. Childhood antibiotic use can increase the susceptibility to IBD because antibiotics disturb the gut microflora, especially in childhood, which is a key period for building the normal balance of the intestinal flora [21–22].

Unlike previous data, we did not confirm the association between IBD and breast feeding. One possible reason for this difference may result from the similar and high proportion of breast feeding among IBD patients (69.5%) and matched controls (83.7%), which may be associated with the lower incidence of IBD in Yunnan Province. Another study in China also indicated that the breast-feeding rate was high [23]. Overall, infancy and childhood factors may influence the composition of the gut microbiome, and this notion is related to the disruption of the intestinal barrier during the onset of IBD.

OCPs have been noted as a risk factor for IBD in the West [24]. Regarding NSAIDs use, a prospective cohort study in the United States confirmed that female patients who used NSAIDs more than 15 days per month had a high risk of developing CD and UC, but these findings did not apply to aspirin use [25]. Minimal research has been performed on prior use of OCPs and NSAIDs in Asia. According to our study, using NA-NSAIDs within 1 month before diagnosis may predispose individuals to UC development but not CD. However, this risk may exist in some patients who have taken NSAIDs for their IBD symptoms. OCP use showed no statistical significance for IBD. However, OCP use may be clinically significant, given that the number of OCP users was low among female participants; only approximately 2% of women used OCPs in Yunnan Province. In 2010, the birth control pill was the first choice of only 1% of married women in China [26], whereas approximately 89% of white women have used it in the United States [27]. This low rate of OCP use may have a protective effect against IBD in Yunnan Province. However, large prospective cohort data regarding the association between dietary factors and IBD are lacking. A systematic review of the literature from Western countries, such as the United States and Canada, and Asian countries, including Japan, indicated that a diet low in vegetables and high in total fats, meat and omega-6 fatty acids may increase the risk of developing IBD [28]. Another study from Japan demonstrated that a high intake of carbohydrates may be a risk factor for IBD [29]. We analyzed greater than 10 items regarding dietary factors and concluded that some were associated with UC onset, such as frequent irregular meal times (OR: 2.287) and consumption of fried foods (OR: 1.920) and frozen dinners (OR: 1.868). Regarding CD, irregular meal times may also be associated with an elevated risk of developing disease (OR: 1.876). The effect of dietary modifications was more obvious in UC patients compared with CD patients, and this difference may be due to the smaller sample size of CD patients. One previous study in Asia indicated that the consumption of tea may reduce the risk of developing CD and UC [5]. Our study revealed that the consumption of tea may serve as an independent protective factor against UC development, whereas a similar conclusion was not reached for CD. Drinking tea is a novel protective factor for IBD development, and studies have confirmed that dietary polyphenols, such as green tea polyphenols, possess both protective and therapeutic effects in the management of IBD [30].

A study from Spain revealed that city living, high education levels and social class at birth are risk factors for developing UC and CD [12]. In our study, no significant association was observed between the degree of education and the risk of IBD. However, mental labor was a risk factor for the development of UC (OR: 2.013), while no association was noted between occupation type and the development of CD.

Moreover, multivariate logistic regression analyses confirmed the association between UC and allergic diseases. Individuals with allergies showed a 5.36-fold increased risk of developing UC compared to those without allergies (OR: 5.361), which may be associated with abnormal immune reactions and provides clues regarding the etiology of IBD. Available data on the effect of allergies on predisposing patients to IBD onset are limited. Physical activity is another valuable factor that has been suggested to provide protection against the onset of IBD. Indeed, physical activity was identified as an independent protective factor against the development of UC and CD, which may be related to its effects on immunomodulation. Lerebours et al. reported that depression is associated with CD; however, it was not identified as an independent risk factor for IBD [29]. Our results revealed that high work stress may be a risk factor for UC (OR: 1.732), and alterations in the gut microbiota could be associated with increased stress levels [31].

Given that only 5 UC cases and no CD cases showed a positive family history of disease, the item “family history” was excluded from data analysis. Few IBD cases in Asia have reported a positive family history [32]. However, a positive family history is a significant factor for IBD and requires further research, including genetic analysis, in Asian patients.

The present study had several limitations. One limitation involved the decision to divide regions to investigate environmental factors of UC and CD. As mentioned for our cohort population, the crude incidence rate of UC and CD is quite different across different regions of China. A total of 1,281 patients with UC and only 59 with CD were enrolled between 1998 and 2007. Due to the limited CD data, the cohort population for CD included permanent residents in 16 cities in Yunnan Province, whereas the cities with the highest incidence rates were used for UC. Given the small sample size for CD cases, some significant findings may have been due to chance as a result of multiple comparison errors. We plan to enlarge the CD sample size in the following study to verify the positive results that have been previously obtained and provide more reliable conclusions. Second, to objectively report the results of the study, selection bias may not have been avoided. In particular, some items were aimed at assessing conditions in childhood. To reduce recall bias, we introduced an ‘unsure’ option to prevent guessing. The rate of ‘unsure’ responses was relatively high (approximately 20%) for questions related to immunizations and antibiotic use. Fortunately, the rate was low for most items (less than 10%). The rates of ‘unsure’ responses were compared between cases and controls, and no significant difference was noted.

In conclusion, the present study is the first nested case-control study to identify environmental factors associated with the subsequent development of IBD in Yunnan Province, which is located in the less-developed southwestern part of China and serves as the home to 25 ethnic minorities. Our study consistently established that environment plays an important role in modulating the subsequent risk of developing IBD. We identified a high proportion of breast-feeding and very low OCP use in participants (both patients and controls in Yunnan Province) that may be associated with a lower incidence of IBD. This finding was unlike previous data from other Asia-Pacific countries and much of the developed world. Dietary factors and irregular meal times were also associated with UC and CD onset, and individuals with allergies showed a higher risk of developing UC than those without allergies. Physical activity was another protective factor for IBD. One factor alone cannot explain disease variation between Asia and the West; however, these factors together may alter the gut microbiome or disturb the host immune system, thus leading to IBD flare-ups [31, 33].

Defining environmental risk factors that could have similar or different effects on CD and UC may help reduce the incidence of these diseases and provide important clues regarding the pathogenesis of IBD.

## Supporting Information

S1 File Questionnaire. (DOCX)

S1 Table Univariate analysis of UC and controls. (DOCX)

S2 Table Univariate analysis of CD and controls. (DOCX)
