# Supplementary material for: Identification of Environmental Factors Associated with Inflammatory Bowel Disease in a Southwestern Highland Region of China: A Nested Case-Control Study
Source: PLoS One. 2016 Apr 12;11(4):e0153524. doi: 10.1371/journal.pone.0153524 (PMC4829194; doi:10.1371/journal.pone.0153524)
Supplement: S1 File — (DOCX) [file pone.0153524.s001.docx]

**S1 File. Questionnaire**

**Number:** **Date:**

[**Hospital**](http://cn.bing.com/dict/clientsearch?mkt=zh-CN&setLang=zh&form=BDVEHC&ClientVer=BDDTV3.5.0.4311&q=%E7%A1%AE%E8%AF%8A%E5%8C%BB%E9%99%A2) **for** [**diagnosis**](http://cn.bing.com/dict/clientsearch?mkt=zh-CN&setLang=zh&form=BDVEHC&ClientVer=BDDTV3.5.0.4311&q=%E7%A1%AE%E8%AF%8A%E5%8C%BB%E9%99%A2)**:**

**Diagnosis:**

口Ulcerative colitis(UC) 口Crohn’s disease(CD) 口IBD-undetermined

**Please fill in the following questions:**

Do you have any other significant health issues or medical conditions not outlined or

mentioned on this form? *(Complete additional questionnaires, as indicated)*

口 Condition(s) 　 List treatment and current status

口 NO other medical conditions or health issues.

**Please fill in some basic information:**

1. **Name:**
2. **Sex:** 口 Male 口 Female
3. **Age:** (ys)
4. **Height:**  (cm)
5. **Weight:**  (kg)
6. **Telephone number:**
7. **E-mail:**
8. **Native place:**
9. **Address:**
10. **Medicare:**
11. **Nationality:** 口 Han 口 Ethnic minority,
12. **Education level:** 口Primary school 口Secondary school 口University
13. **Occupational type:** 口 Workers 口 Peasants 口 Professionals**^※^**

口 Public Servants 口 Freelancers 口 Others**^※※^**.

*(***^※^***Professionals include teachers, doctors, nurses, etc.* **^※※^***Others include  housewives, retired people, job waiting people,  underemployed workers, etc. )*

14. **Marital status:** 口 Unmarried口Married口Divorced口Widowed

15. **Please fill in some items about working and living environment:**

15.1 **Labor type:** 口 Manual labor 口 Mixed 口 Mental labor

*(**People who do manual labor include unskilled manual workers, like construction workers, as well as farm and forestry workers. People who do mental labor include desk jobs, like clerical workers, managers, higher administrators, scientific research-related workers, and clerical employees. Mixed labor include some skilled and specialised work required more manual labor, like dentists, drivers, seamen, cooks.)*

15.2 **Work stress:** 口 Never 口 General 口 High

16. **Residence in recent 5-years:** 口 Countryside 口 Town 口 City

17. **Average living space:** ㎡, 口<30 ㎡口≥30 ㎡

18. **Housing type:** 口 High buildings 口 Short brick house 口 Adobe house

**Some eating habits and lifestyle prior to diagnosis:**

1. **Irregular meal times:** 口 Never 口1-2 times /week 口≥3 times /week

2. **Eating meat:** 口 Never 口1-2 times /week 口≥3 times /week

3. **Eating eggs:** 口 Never 口1-2 times /week 口≥3 times /week

4. **Consumption of milk:** 口 Never 口1-2 times /week 口≥3 times /week

5. **Eating fried foods:** 口 Never 口1-2 times /week 口≥3 times /week

6. **Eating salty foods** *(bacon, salted fish, pickled mustard green, etc.)***:**

口 Never 口1-2 times /week 口≥3 times /week

7. **Eating spicy foods:** 口 Never 口1-2 times /week 口≥3 times /week

8. **Consumption of sugars and sweets:**

口 Never 口1-2 times /week 口≥3 times /week

9. **Fish intake:** 口Never口1-2 times /week 口≥3 times /week

10. **Frozen dinners intake:** 口Never口1-2 times /week口≥3 times /week

11. **Vegetables intake:** 口Never口1-2 times /week口≥3 times /week

12. **Consumption of fruits:** 口Never口1-2 times /week口≥3 times /week

13. **Drinking water:**

口 Well water-based 口 Tap water-based 口 Boiled water-based 口 Mineral water-based

14. **Diet composition:** 口 Vegetable-based 口 Mixed meals 口 Meat-based

15. **Consumption of tea** *(If no, skip this item; If yes, please fill in 15.1and 15.2)***:**

口 No 口 Yes

15.1  **Frequency of tea consumption：**口1-2 times /week口≥3 times /week

15.2 **Main types of tea:** 口 Black tea 口 Green tea 口 Scented tea 口 Others,

*(Green tea is pale, greenish yellow, and the black tea is a deep amber color.)*

16. **Smoking** *(If never, skip this item; If current, please fill in 16.1and 16.2)***:**

口 Never smoking 口 Current smoking 口 Ex- smoking: (months)

16.1 **Average number of cigarettes smoked per day:**

口<10 cigarettes 口10-20 cigarettes 口>20 cigarettes

16.2 **Duration of smoking:**

口<1year口1-5 years口5-10 years口≥10 years

*(Current smoking was defined as smoking at least one cigarette/day, ex-smoking as reporting having smoked at least one cigarette/day but having quit, and never smoking as never having smoked one cigarette/day.)*

17. **Drinking** *(If no, skip this item; If yes, please fill in 17.1, 17.2 and 17.3*):

口No 口Yes

*(Drinking is defined as more than once of alcohol drinking per month.)*

17.1 **Frequency of drinking:**

口1-2 times /month 口1-2 times /week 口≥3 times/week

17.2 **Type of alcohol:**

口 White wine 口 Red wine 口 Beer 口 Others:

17.3 **Consumption of alcohol:**

Amount of alcohol every time: (ml); Degrees: ;

Time length:  **(**ys)

18. **Physical activity:**

口 Never 口1-2 times /week 口≥3 times/week

*(Physical activity is any rhythmic and continuous activity more than 20 minutes at a time).*

19. **Mean sleep duration:** 口＜6 hours口≥6 hours

**Other items prior to the illness onset:**

1. **Family history** *(parent or siblings had Ulcerative colitis or Crohn’s disease)***:**

口 No 口 Yes

2. **Allergies:** 口 No 口 Yes,

3. **Pet ownership:** 口 No 口 Yes,

4. **Appendectomy:** 口 No 口 Yes

5. **Breast-feeding:** 口 Never 口﹤3 months 口≥3 months 口 Unsure

6. **Delivery mode:** 口 Natural birth 口 Cesarean

7. **Childhood antibiotic use (before 14 years):**

口 Never 口 1-2 times /year 口 ≥3 times/year 口 Unsure

8. **Childhood gastrointestinal infections (before 14 years):**

口 Never 口 1-2 times /year 口 ≥3 times/year 口 Unsure

9. **Immunizations or infections measles, rubella, mumps (before 14 years):**

口 No 口 Yes, 口 Unsure

10. **Non-aspirin non-steroidal anti-inflammatory drugs (NA-NSAIDs) intake** *(ibuprofen, diclofenac, etc.)***:**

口 Never 口＜1 month 口≥1 month

11. **Aspirin intake:** 口 Never 口 ＜1 month 口 ≥1 month

12. **Oral contraceptive use:** 口 Never 口 Past 口Current,＜5 years 口Current,≥5 years

13. **Parasitic infection:**口 Never 口 Past 口 Uns
