# Supplementary material for: Identification of Environmental Factors Associated with Inflammatory Bowel Disease in a Southwestern Highland Region of China: A Nested Case-Control Study
Source: PLoS One. 2016 Apr 12;11(4):e0153524. doi: 10.1371/journal.pone.0153524 (PMC4829194; doi:10.1371/journal.pone.0153524)
Supplement: S1 Table — (DOCX) [file pone.0153524.s002.docx]

**S1 table. Univariate analysis of UC and controls**

| **Variables** | **UC**  **(678)** | | **UC controls**  **(2712)** | | **OR** | **95%CIs** | ***P*** |
| --- | --- | --- | --- | --- | --- | --- | --- |
| **Sex:** | |  |  |  | |  |  |
| Female (%) | | 306 (45.1) | 1224 (45.1) | 1 | |  |  |
| Male (%) | | 372 (54.9) | 1488(54.9) | 1.00 | | 0.84–1.18 | 1.000 |
| **Nationality：** | |  |  |  | |  |  |
| Han (%) | | 665(98.1) | 2657(98.0) | 1 | |  |  |
| Minority (%) | | 13 (1.9) | 55 (2.0) | 1.06 | | 0.58–1.95 | 0.854 |
| **Education level：** | |  |  |  | |  |  |
| Primary school (%) | | 212 (31.3) | 935 (34.5) | 1 | |  |  |
| Secondary school (%) | | 261 (38.5) | 941 (34.7) | 0.82 | | 0.67–1.00 | 0.051 |
| University (%) | | 205 (30.2) | 836 (30.8) | 0.93 | | 0.75–1.15 | 0.472 |
| **Allergies：** | |  |  |  | |  |  |
| Yes (%) | | 146 (21.5) | 222 (8.2) | **3.08** | | **2.45–3.87** | **<0.001** |
| No (%) | | 532 (78.5) | 2490 (91.8) | 1 | |  |  |
| **Pet ownership：** | |  |  |  | |  |  |
| Yes (%) | | 185 (27.3) | 667 (24.6) | 1.15 | | 0.95–1.39 | 0.148 |
| No (%) | | 493 (72.7) | 2045 (75.4) | 1 | |  |  |
| **Appendectomy：** | |  |  |  | |  |  |
| Yes (%) | | 38 (5.6) | 122 (4.5) | 1.26 | | 0.87–1.83 | 0.224 |
| No (%) | | 640 (94.4) | 2590 (95.5) | 1 | |  |  |
| **Breast-feeding：** | |  |  |  | |  |  |
| Never (%) | | 43 (6.3) | 176 (6.5) | 1 | |  |  |
| **<**3 months (%) | | 18 (2.7) | 122 (4.5) | 0.64 | | 0.33–1.10 | 0.095 |
| ≥3 months (%) | | 453 (66.8) | 2147 (79.2) | 0.86 | | 0.61–1.22 | 0.409 |
| **Delivery mode：** | |  |  |  | |  |  |
| Cesarean (%) | | 78(11.5) | 338(12.5) | 1 | |  |  |
| Natural birth (%) | | 600(88.5) | 2374(87.5) | 1.10 | | 0.84–1.42 | 0.496 |
| **Childhood antibiotic use:** | |  |  |  | |  |  |
| Never (%) | | 256(37.8) | 1004(37.0) | 1 | |  |  |
| 1-2 times /year (%) | | 156(23.0) | 650(24.0) | 0.94 | | 0.75–1.18 | 0.593 |
| ≥3 times/year (%) | | 59(8.7) | 104(3.8) | **2.23** | | **1.57–3.15** | **<0.001** |
| **Childhood intestinal infections:** | |  |  |  | |  |  |
| Never (%) | | 309(45.6) | 1345(49.6) | 1 | |  |  |
| 1-2 times /year (%) | | 132(19.5) | 323(11.9) | **1.78** | | **1.40–2.26** | **<0.001** |
| ≥3 times/year (%) | | 29(4.3) | 146(5.4) | 0.87 | | 0.57–1.31 | 0.494 |
| **Immunizations:** | |  |  |  | |  |  |
| No (%) | | 496(71.2) | 1871(69.0) | 1 | |  |  |
| Yes (%) | | 97(14.3) | 376(13.9) | 0.97 | | 0.76-1.24 | 0.827 |
| **NA-NSAIDs intake:** | |  |  |  | |  |  |
| Never (%) | | 612(90.3) | 2546(93.9) | 1 | |  |  |
| **<**1 month (%) | | 49(7.2) | 100(3.7) | **2.04** | | **1.43–2.90** | **<0.001** |
| ≥ 1 month (%) | | 17(2.5) | 58(2.2) | 1.22 | | 0.71–2.11 | 0.477 |
| **Aspirin intake:** | |  |  |  | |  |  |
| Never (%) | | 660(97.3) | 2659(98.0) | 1 | |  |  |
| **<**1 month (%) | | 8(1.2) | 29(1.1) | 1.11 | | 0.51–2.44 | 0.793 |
| ≥ 1 month (%) | | 10(1.5) | 24(0.9) | 1.68 | | 0.80–3.53 | 0.167 |
| **OCP use:** | |  |  |  | |  |  |
| Never (%) | | 234(76.5) | 1005(82.1) | 1 | |  |  |
| Past (%) | | 65(21.2) | 200(16.4) | 1.40 | | 1.02–1.91 | 0.037 |
| Current, **<**5 years(%) | | 6(2.0) | 16(1.3) | 1.61 | | 0.62–4.16 | 0.321 |
| Current, ≥5 years(%) | | 1(0.3) | 3(0.2) | 1.43 | | 0.15–13.83 | 1.000 |
| **Parasitic infection:** | |  |  |  | |  |  |
| Never (%) | | 584(86.1) | 2390(88.1) | 0.84 | | 0.65–1.07 | 0.158 |
| Past (%) | | 94(13.9) | 322(11.9) | 1 | |  |  |
| **Average living space:** | |  |  |  | |  |  |
| **<**30 ㎡(%) | | 172(25.4) | 790(29.1) | 0.83 | | 0.68–1.00 | 0.052 |
| ≥30 ㎡(%) | | 506(74.6) | 1922(70.9) | 1 | |  |  |
| **Housing type:** | |  |  |  | |  |  |
| High buildings (%) | | 577(85.1) | 2236(82.4) | 1 | |  |  |
| Short brick house (%) | | 93(13.7) | 427(15.7) | 0.84 | | 0.66–1.08 | 0.170 |
| Adobe house (%) | | 8(1.2) | 49(1.8) | 0.63 | | 0.30–1.34 | 0.229 |
| **Labor type:** | |  |  |  | |  |  |
| Manual labor (%) | | 218(32.2) | 1132(41.7) | 1 | |  |  |
| Mixed (%) | | 177(26.1) | 788(29.1) | 1.17 | | 0.94–1.45 | 0.167 |
| Mental labor (%) | | 283(41.7) | 792(29.2) | **1.86** | | **1.52–2.26** | **<0.001** |
| **Work stress:** | |  |  |  | |  |  |
| Never (%) | | 161(23.8) | 810(29.9) | 1 | |  |  |
| General (%) | | 344(50.7) | 1436(52.9) | 1.21 | | 0.98–1.48 | 0.076 |
| High (%) | | 173(25.5) | 466(17.2) | **1.87** | | **1.47–2.38** | **<0.001** |
| **Irregular meal times:** | |  |  |  | |  |  |
| Never (%) | | 475(70.0) | 2017(74.4) | 1 | |  |  |
| 1-2 times /week (%) | | 71(10.5) | 405(14.9) | 0.74 | | 0.57–0.98 | 0.032 |
| ≥3 times/week (%) | | 132(19.5) | 290(10.7) | **1.93** | | **1.54–2.43** | **<0.001** |
| **Meat:** | |  |  |  | |  |  |
| Never (%) | | 6(0.9) | 13(0.5) | 1 | |  |  |
| 1-2 times /week (%) | | 112(16.5) | 547(20.2) | 0.44 | | 0.17–1.19 | 0.098 |
| ≥3 times/week (%) | | 560(82.6) | 2152(70.4) | 0.56 | | 0.21–1.49 | 0.375 |
| **Eggs:** | |  |  |  | |  |  |
| Never (%) | | 179(26.4) | 661(24.4) | 1 | |  |  |
| 1-2 times /week (%) | | 341(50.3) | 1477(54.5) | 0.85 | | 0.70–1.04 | 0.123 |
| ≥3 times/week (%) | | 158(23.3) | 574(21.2) | 1.02 | | 0.80–1.29 | 0.895 |
| **Milk:** | |  |  |  | |  |  |
| Never (%) | | 407(60.0) | 1508(55.6) | 1 | |  |  |
| 1-2 times /week (%) | | 166(24.5) | 747(27.5) | 0.82 | | 0.67–1.01 | 0.057 |
| ≥3 times/week (%) | | 105(15.5) | 457(16.9) | 0.85 | | 0.67–1.08 | 0.186 |
| **Fried foods:** | |  |  |  | |  |  |
| Never (%) | | 175(25.8) | 815(30.1) | 1 | |  |  |
| 1-2 times /week (%) | | 370(54.6) | 1568(57.8) | 1.10 | | 0.90–1.34 | 0.352 |
| ≥3 times/week (%) | | 133(19.6) | 329(12.1) | **1.88** | | **1.45–2.44** | **<0.001** |
| **Salty foods:** | |  |  |  | |  |  |
| Never (%) | | 127(18.7) | 567(20.9) | 1 | |  |  |
| 1-2 times /week (%) | | 367(54.1) | 1673(61.7) | 0.98 | | 0.78–1.22 | 0.855 |
| ≥3 times/week (%) | | 184(27.1) | 472(17.4) | **1.74** | | **1.35–2.25** | **<0.001** |
| **Spicy foods:** | |  |  |  | |  |  |
| Never (%) | | 103(15.2) | 356(13.7) | 1 | |  |  |
| 1-2 times /week (%) | | 279(41.2) | 1098(40.5) | 0.88 | | 0.68–1.13 | 0.319 |
| ≥3 times/week (%) | | 296(43.6) | 1258(46.4) | 0.81 | | 0. 63–1.05 | 0.109 |
| **Consumption of sweets:** | |  |  |  | |  |  |
| Never (%) | | 151(22.3) | 597(22.0) | 1 | |  |  |
| 1-2 times /week (%) | | 341(50.3) | 1470(54.2) | 0.92 | | 0.74–1.14 | 0.428 |
| ≥3 times/week (%) | | 186(27.4) | 645(23.8) | 1.14 | | 0.90–1.45 | 0.228 |
| **Fish:** | |  |  |  | |  |  |
| Never (%) | | 376(55.5) | 1435(52.9) | 1 | |  |  |
| 1-2 times /week (%) | | 276(40.7) | 1150(42.4) | 0.92 | | 0.77–1.09 | 0.322 |
| ≥3 times/week (%) | | 26(3.8) | 127(4.7) | 0.78 | | 0.51–1.21 | 0.267 |
| **Frozen dinners:** | |  |  |  | |  |  |
| Never (%) | | 164(24.2) | 812(29.9) | 1 | |  |  |
| 1-2 times /week (%) | | 211(31.1) | 969(35.7) | 1.08 | | 0.86–1.35 | 0.511 |
| ≥3 times/week (%) | | 303(44.7) | 931(34.3) | **1.61** | | **1.30–1.99** | **<0.001** |
| **Vegetables:** | |  |  |  | |  |  |
| 1-2 times /week (%) | | 64(9.4) | 297(11.0) | 0.85 | | 0.64–1.13 | 0.254 |
| ≥3 times/week (%) | | 614(90.6) | 2415(89.0) | 1 | |  |  |
| **Fruits:** | |  |  |  | |  |  |
| Never (%) | | 78(11.5) | 193(7.1) | 1 | |  |  |
| 1-2 times /week (%) | | 202(29.8) | 867(32.0) | **0.58** | | **0.43–0.78** | **<0.001** |
| ≥3 times/week (%) | | 398(58.7) | 1652(60.9) | **0.60** | | **0.45–0.79** | **<0.001** |
| **Drinking water:** | |  |  |  | |  |  |
| Well water-based (%) | | 30(4.4) | 68(2.5) | 1 | |  |  |
| Tap water-based (%) | | 55(8.1) | 218(8.0) | **0.57** | | **0.34–0.96** | **0.034** |
| Boiled water-based (%) | | 243(35.8) | 948(35.0) | **0.58** | | **0.37–0.91** | **0.017** |
| Mineral water-based (%) | | 350(51.6) | 1478(54.5) | **0.54** | | **0.34–0.84** | **0.005** |
| **Diet composition:** | |  |  |  | |  |  |
| Vegetable-based (%) | | 54(8.0) | 210(7.7) | 1 | |  |  |
| Mixed meals (%) | | 590(87.0) | 2298(84.7) | 1.00 | | 0.73–1.37 | 0.992 |
| Meat-based (%) | | 34(5.0) | 204(7.5) | 0.65 | | 0.41–1.04 | 0.070 |
| **Consumption of tea:** | |  |  |  | |  |  |
| Yes (%) | | 222(32.7) | 1308(48.2) | **0.52** | | **0.44–0.62** | **<0.001** |
| No (%) | | 456(67.3) | 1404(51.8) | 1 | |  |  |
| **Frequency of tea consumption :** | |  |  |  | |  |  |
| 1-2 times /week (%) | | 90(40.5) | 554(42.4) | 0.93 | | 0.70–1.24 | 0.613 |
| ≥3 times /week (%) | | 132(59.5) | 754(57.6) | 1 | |  |  |
| **Smoking:** | |  |  |  | |  |  |
| No-current (%) | | 524 (77.3) | 1930(71.2) | 1 | |  |  |
| Current (%) | | 120(17.7) | 673(24.8) | **0.68** | | **0.53–0.82** | **<0.001** |
| Ex-smoking (%) | | 34（5.0） | 109（4.0） | 1.15 | | 0.77–1.71 | 0.493 |
| **Amount of smoking:** | |  |  |  | |  |  |
| **<**10 /day (%) | | 29(24.0) | 147(21.8) | 1 | |  |  |
| 10-20 /day (%) | | 70(57.9) | 385(57.1) | 0.92 | | 0.57–1.48 | 0.735 |
| >20 /day (%) | | 22(18.2) | 142(21.1) | 0.79 | | 0.43–1.43 | 0.429 |
| **Alcohol drinking:** | |  |  |  | |  |  |
| Yes (%) | | 161(23.7) | 671(24.7) | 1 | |  |  |
| Never (%) | | 517(76.3) | 2041(75.3) | 1.06 | | 0.88–1.29 | 0.590 |
| **Frequency of drinking:** | |  |  |  | |  |  |
| 1-2 times /month (%) | | 67(41.6) | 194(28.9) | 1 | |  |  |
| 1-2 times /week (%) | | 50(31.1) | 269(40.1) | 0.54 | | 0.36–0.81 | 0.003 |
| ≥3 times/week (%) | | 44(27.3) | 208(31.0) | 0.61 | | 0.40–0.94 | 0.024 |
| **Physical activity:** | |  |  |  | |  |  |
| Never (%) | | 209(30.8) | 558(20.6) | 1 | |  |  |
| 1-2 times /week (%) | | 282(41.6) | 1182(43.6) | **0.64** | | **0.52–0.78** | **<0.001** |
| ≥3 times/week (%) | | 187(27.6) | 972(35.8) | **0.51** | | **0.41–0.64** | **<0.001** |
| **Mean sleep duration:** | |  |  |  | |  |  |
| **<**6 hours (%) | | 147(21.7) | 491(18.1) | **1.25** | | **1.02–1.54** | **0.033** |
| ≥6 hours (%) | | 531(78.3) | 2221(81.9) | 1 | |  |  |
