# Supplementary material for: Identification of Environmental Factors Associated with Inflammatory Bowel Disease in a Southwestern Highland Region of China: A Nested Case-Control Study
Source: PLoS One. 2016 Apr 12;11(4):e0153524. doi: 10.1371/journal.pone.0153524 (PMC4829194; doi:10.1371/journal.pone.0153524)
Supplement: S2 Table — (DOCX) [file pone.0153524.s003.docx]

**S2 table. Univariate analysis of CD and controls**

| **Variables** | **CD**  **(102)** | | **CD controls**  **(410)** | | **OR** | **95%CI** | ***p*** |
| --- | --- | --- | --- | --- | --- | --- | --- |
| **Sex:** | |  |  |  | |  |  |
| Female (%) | | 36 (35.3) | 145 (35.4) | 1 | |  |  |
| Male (%) | | 66 (64.7) | 265 (64.6) | 1.00 | | 0.64–1.58 | 0.989 |
| **Nationality:** | |  |  |  | |  |  |
| Han (%) | | 98(96.1) | 391(95.4) |  | |  |  |
| Minority (%) | | 4 (3.9) | 19 (4.6) | 1.19 | | 0.40–3.58 | 0.965 |
| **Education level:** | |  |  |  | |  |  |
| Primary school (%) | | 30 (29.4) | 134 (32.7) | 1 | |  |  |
| Secondary school (%) | | 40 (39.2) | 154 (37.5) | 1,16 | | 0.69–1.97 | 0.580 |
| University (%) | | 32 (31.4) | 122 (29.8) | 1.17 | | 0.67–2.04 | 0.576 |
| **Allergies:** | |  |  |  | |  |  |
| Yes (%) | | 13 (12.7) | 27 (6.6) | **2.07** | | **1.03–4.18** | **0.038** |
| No (%) | | 89 (87.3) | 383 (93.4) |  | |  |  |
| **Pet ownership:** | |  |  |  | |  |  |
| Yes (%) | | 27 (26.5) | 99 (24.1) | 1.13 | | 0.69–1.86 | 0.626 |
| No (%) | | 75 (73.5) | 311 (75.9) |  | |  |  |
| **Appendectomy:** | |  |  |  | |  |  |
| Yes (%) | | 14 (13.7) | 23 (5.6) | **2.68** | | **1.33–5.41** | **0.005** |
| No (%) | | 88 (86.3) | 387 (94.4) |  | |  |  |
| **Breast-feeding:** | |  |  |  | |  |  |
| Never (%) | | 10 (9.8) | 31 (7.6) | 1 | |  |  |
| <3 months (%) | | 11 (10.8) | 28 (6.8) | 1.22 | | 0.45–3.30 | 0.698 |
| ≥3 months (%) | | 72 (70.6) | 314 (76.6) | 0.70 | | 0.33–1.50 | 0.375 |
| **Delivery mode:** | |  |  |  | |  |  |
| Cesarean (%) | | 12 (11.8) | 38 (9.3) |  | |  |  |
| Natural birth (%) | | 90 (88.2) | 372 (90.7) | 0.77 | | 0.39–1.53 | 0.447 |
| **Childhood antibiotic use:** | |  |  |  | |  |  |
| Never (%) | | 39 (38.2) | 144 (35.1) | 1 | |  |  |
| 1-2 times /year (%) | | 38 (37.3) | 119 (29.0) | 1.18 | | 0.71–1.96 | 0.525 |
| ≥3 times/year (%) | | 8 (7.8) | 23 (5.6) | 1.28 | | 0.53–3.09 | 0.576 |
| **Childhood intestinal infections:** | |  |  |  | |  |  |
| Never (%) | | 45 (44.1) | 190 (46.3) | 1 | |  |  |
| 1-2 times /year (%)* | | 29 (28.4) | 68 (16.6) | 1.80 | | 1.05–3.10 | 0.052 |
| ≥3 times/year (%) | | 6 (5.9) | 18 (4.4) | 1.41 | | 0.53–3.75 | 0.677 |
| **Immunizations:** | |  |  |  | |  |  |
| No (%) | | 73 (71.6) | 284 (69.3) | 1.01 | | 0.53–1.92 | 0.976 |
| Yes (%) | | 14 (13.7) | 55 (13.4) |  | |  |  |
| **NA-NSAIDs intake:** | |  |  |  | |  |  |
| Never (%) | | 94 (92.2) | 373 (91.0) | 1 | |  |  |
| <1 month (%) | | 6 (5.9) | 25 (6.1) | 0.95 | | 0.38–2.39 | 0.917 |
| ≥ 1 month (%) | | 2 (2.0) | 12 (2.9) | 0.66 | | 0.15–3.01 | 0.842 |
| **Aspirin intake:** | |  |  |  | |  |  |
| Never (%) | | 34 (94.4) | 136 (93.8) | 1 | |  |  |
| <1 month (%) | | 2 (5.6) | 9 (6.2) | 0.89 | | 0.18–4.31 | 0.884 |
| ≥ 1 month (%) | | 0 | 0 |  | |  |  |
| **Parasitic infection:** | |  |  |  | |  |  |
| Never (%) | | 95 (93.1) | 369 (90.0) | 1.51 | | 0.66–3.47 | 0.331 |
| Past (%) | | 7 (6.9) | 41 (10.0) |  | |  |  |
| **Average living space:** | |  |  |  | |  |  |
| <30㎡(%) | | 31 (30.4) | 117 (28.5) | 1.09 | | 0.68–1.76 | 0.711 |
| ≥30㎡(%) | | 71 (69.6) | 293 (71.5) |  | |  |  |
| **Housing type:** | |  |  |  | |  |  |
| High buildings (%) | | 89 (87.3) | 349 (85.1) | 1 | |  |  |
| Short brick house (%) | | 12 (11.8) | 55 (13.4) | 0.86 | | 0.44–1.67 | 0.646 |
| Adobe house (%) | | 1 (1.0) | 6 (1.5) | 0.65 | | 0.08–5.50 | 1.000 |
| **Labor type:** | |  |  |  | |  |  |
| Manual labor (%) | | 41 (40.2) | 161 (39.3) | 1 | |  |  |
| Mixed (%) | | 28 (27.5) | 123 (30.0) | 0.89 | | 0.52–1.53 | 0.681 |
| Mental labor (%) | | 33 (32.4) | 126 (30.7) | 1.03 | | 0.62–1.72 | 0.915 |
| **Work stress:** | |  |  |  | |  |  |
| Never (%) | | 27 (26.5) | 100 (24.4) | 1 | |  |  |
| General (%) | | 51 (50.0) | 232 (56.6) | 0.81 | | 0.48–1.37 | 0.440 |
| High (%)* | | 24 (23.5) | 78 (19.0) | 1.14 | | 0.61–2.13 | 0.682 |
| **Irregular meal times:** | |  |  |  | |  |  |
| Never (%) | | 53 (52.0) | 267 (65.1) | 1 | |  |  |
| 1-2 times /week (%) | | 18 (17.6) | 68 (16.6) | 1.33 | | 0.73–2.42 | 0.344 |
| ≥3 times/week (%) | | 31 (30.4) | 75 (18.3) | **2.08** | | **1.25–3.47** | **0.004** |
| **Meat:** | |  |  |  | |  |  |
| Never (%) | | 0 (0.0) | 1 (0.2) | - | | - | - |
| 1-2 times /week (%) | | 17 (16.7) | 79 (19.3) | 1 | |  |  |
| ≥3 times/week (%) | | 85 (83.3) | 330 (80.5) | 1.20 | | 0.67–2.13 | 0.540 |
| **Eggs:** | |  |  |  | |  |  |
| Never (%) | | 28 (27.5) | 116 (28.3) | 1 | |  |  |
| 1-2 times /week (%) | | 52 (51.0) | 201 (49.0) | 1.07 | | 0.64–1.79 | 0.791 |
| ≥3 times/week (%) | | 22 (21.6) | 93 (22.7) | 0.98 | | 0.53–1.82 | 0.949 |
| **Milk:** | |  |  |  | |  |  |
| Never (%) | | 62 (60.8) | 241 (58.8) | 1 | |  |  |
| 1-2 times /week (%) | | 22 (21.6) | 100 (24.4) | 0.86 | | 0.50–1.47 | 0.569 |
| ≥3 times/week (%) | | 18 (17.6) | 69 (16.8) | 1.01 | | 0.56–1.83 | 0.963 |
| **Fried foods:** | |  |  |  | |  |  |
| Never (%) | | 41 (40.2) | 155 (37.8) | 1 | |  |  |
| 1-2 times /week (%) | | 49 (48.0) | 211 (51.5) | 0.88 | | 0.55–1.40 | 0.582 |
| ≥3 times/week (%) | | 12 (11.8) | 44 (10.7) | 1.03 | | 0.50–2.13 | 0.934 |
| **Salty foods:** | |  |  |  | |  |  |
| Never (%) | | 15 (14.7) | 67 (16.3) | 1 | |  |  |
| 1-2 times /week (%) | | 60 (58.8) | 258 (62.9) | 1.04 | | 0.56–1.94 | 0. 905 |
| ≥3 times/week (%) | | 27 (26.5) | 85 (20.7) | 1.42 | | 0.70–2.88 | 0.331 |
| **Spicy foods:** | |  |  |  | |  |  |
| Never (%) | | 18 (17.6) | 61 (14.9) | 1 | |  |  |
| 1-2 times /week (%) | | 44 (43.1) | 174 (42.4) | 0.86 | | 0.46–1,60 | 0.626 |
| ≥3 times/week (%) | | 40 (39.2) | 175 (42.7) | 0.78 | | 0.41–1.45 | 0.425 |
| **Sugars and sweets:** | |  |  |  | |  |  |
| Never (%) | | 20 (19.6) | 81 (19.7) | 1 | |  |  |
| 1-2 times /week (%) | | 56 (54.9) | 232 (56.6) | 0.98 | | 0.55–1.73 | 0.938 |
| ≥3 times/week (%) | | 23 (22.5) | 93 (22.7) | 1.00 | | 0.51–1.95 | 0.996 |
| **Fish:** | |  |  |  | |  |  |
| Never (%) | | 52 (51.0) | 212 (51.7) | 1 | |  |  |
| 1-2 times /week (%) | | 41 (40.2) | 170 (41.4) | 1.00 | | 0.63–1.59 | 0.942 |
| ≥3 times/week (%) | | 6 (5.9) | 24 (5.9) | 1.04 | | 0.40–2.68 | 0.968 |
| **Frozen dinners:** | |  |  |  | |  |  |
| Never (%) | | 25 (24.5) | 112 (27.3) | 1 | |  |  |
| 1-2 times /week (%) | | 43 (42.2) | 170 (41.5) | 1.13 | | 0.66–2.00 | 0.654 |
| ≥3 times/week (%) | | 34 (33.3) | 128 (31.2) | 1.19 | | 0.67–2,12 | 0.553 |
| **Vegetables:** | |  |  |  | |  |  |
| 1-2 times /week (%) | | 25 (24.5) | 61 (14.9) | **1.86** | | **0.64–1.13** | **0.020** |
| ≥3 times/week (%) | | 77 (75.5) | 349 (85.1) |  | |  |  |
| **Fruits:** | |  |  |  | |  |  |
| Never (%) | | 5 (4.9) | 19 (4.6) | 1 | |  |  |
| 1-2 times /week (%) | | 52 (51.0) | 142 (34.6) | 1.39 | | 0.49–3.92 | 0.530 |
| ≥3 times/week (%) | | 45 (44.1) | 249 (60.7) | 0.69 | | 0.24–1.93 | 0.672 |
| **Drinking water:** | |  |  |  | |  |  |
| Well water-based (%) | | 0 | 8 (2.0) | - | |  |  |
| Tap water-based (%) | | 5 (4.9) | 23 (5.6) | 1 | |  |  |
| Boiled water-based (%) | | 37 (36.3) | 145 (35.4) | 1.17 | | 0.42–3.30 | 0.761 |
| Mineral water-based (%) | | 60 (58.8) | 234 (57.1) | 1.18 | | 0.43–3.23 | 0.748 |
| **Diet composition:** | |  |  |  | |  |  |
| Vegetable-based (%) | | 5 (4.9) | 32 (7.8) | 1 | |  |  |
| Mixed meals (%) | | 88 (86.3) | 346 (84.4) | 1.63 | | 0.62–4.30 | 0.321 |
| Meat-based (%) | | 9 (8.8) | 32 (7.8) | 1.80 | | 0.54–5.96 | 0.332 |
| **Consumption of tea:** | |  |  |  | |  |  |
| No (%) | | 64 (62.7) | 231 (56.3) | 1.31 | | 0.84–2.04 | 0.242 |
| Yes (%) | | 38 (37.3) | 179 (43.7) |  | |  |  |
| **Frequency of tea consumption:** | |  |  |  | |  |  |
| 1-2 times /week (%) | | 19 (50.0) | 74 (41.3) | 1.42 | | 0.70–2.86 | 0.327 |
| ≥3 times /week (%) | | 19 (50.0) | 105 (58.7) |  | |  |  |
| **Smoking:** | |  |  |  | |  |  |
| No-current (%) | | 75 (73.5) | 294 (71.7) | 1 | |  |  |
| Current (%) | | 22 (21.6) | 96 (23.4) | 0.90 | | 0.53–1.52 | 0.691 |
| Ex-smoking | | 5（4.9） | 20（4.9） | 0.98 | | 0.36–2.70 | 0.969 |
| **Amount of smoking:** | |  |  |  | |  |  |
| <10 /day (%) | | 4 (18.2) | 18(18.8) | 1 | |  |  |
| 10-20 /day (%) | | 15 (68.2) | 60 (62.5) | 1.13 | | 0.33–3.82 | 1.000 |
| >20 /day (%) | | 3 (13.6) | 18 (18.8) | 0.75 | | 0.15–3.84 | 0.729 |
| **Alcohol drinking:** | |  |  |  | |  |  |
| Never (%) | | 79 (77.5) | 323 (78.8) | 0.93 | | 0.55–1.56 | 0.770 |
| Yes (%) | | 23 (22.5) | 87 (21.2) | 1 | |  |  |
| **Frequency of drinking:** | |  |  |  | |  |  |
| 1-2 times /month (%) | | 5 (21.7) | 22 (25.3) | 1 | |  |  |
| 1-2 times /week (%) | | 13 (56.5) | 42 (48.3) | 1.36 | | 0.43–4.32 | 0.599 |
| ≥3 times/week (%) | | 5 (21.8) | 23 (26.4) | 0.96 | | 0.24–3.78 | 1.000 |
| **Physical activity:** | |  |  |  | |  |  |
| Never (%) | | 51 (50.0) | 116 (28.3) | 1 | |  |  |
| 1-2 times /week (%) | | 21 (20.6) | 150 (36.6) | **0.32** | | **0.18–0.56** | **<0.001** |
| ≥3 times/week (%) | | 30 (29.4) | 144 (35.1) | **0.47** | | **0.28–0.79** | **0.004** |
| **Mean sleep duration:** | |  |  |  | |  |  |
| <6 hours (%) | | 14 (13.7) | 62 (15.1) | 0.89 | | 0.48–1.67 | 0.723 |
| ≥6 hours (%) | | 88 (86.3) | 348 (84.9) |  | |  |  |
